# Mapping Function from Dynamics: Future Challenges for Network-Based Models of Protein Structures

**DOI:** 10.3389/fmolb.2021.744646

**Published:** 2021-10-11

**Authors:** Lorenza Pacini, Rodrigo Dorantes-Gilardi, Laurent Vuillon, Claire Lesieur

**Affiliations:** ^1^ Ecole Centrale de Lyon, Ampère, UMR5005, Univ. Lyon, CNRS, INSA Lyon, Université Claude Bernard Lyon 1, Villeurbanne, France; ^2^ Institut Rhônalpin des Systèmes Complexes, IXXI-ENS-Lyon, Lyon, France; ^3^ USMB, CNRS, LAMA UMR5127, Le Bourget du Lac, France

**Keywords:** protein structure, protein dynamics, protein function, network-based models, space occupancy

## Abstract

Proteins fulfill complex and diverse biological functions through the controlled atomic motions of their structures (functional dynamics). The protein composition is given by its amino-acid sequence, which was assumed to encode the function. However, the discovery of functional sequence variants proved that the functional encoding does not come down to the sequence, otherwise a change in the sequence would mean a change of function. Likewise, the discovery that function is fulfilled by a set of structures and not by a unique structure showed that the functional encoding does not come down to the structure either. That leaves us with the possibility that a set of atomic motions, achievable by different sequences and different structures, encodes a specific function. Thanks to the exponential growth in annual depositions in the Protein Data Bank of protein tridimensional structures at atomic resolutions, network models using the Cartesian coordinates of atoms of a protein structure as input have been used over 20 years to investigate protein features. Combining networks with experimental measures or with Molecular Dynamics (MD) simulations and using typical or ad-hoc network measures is well suited to decipher the link between protein dynamics and function. One perspective is to consider static structures alone as alternatives to address the question and find network measures relevant to dynamics that can be subsequently used for mining and classification of dynamic sequence changes functionally robust, adaptable or faulty. This way the set of dynamics that fulfill a function over a diversity of sequences and structures will be determined.

## Introduction

Proteins fulfill complex and diverse biological functions through the controlled atomic motions of their structures (Wingert et al., 2021). The protein composition is given by its amino-acid sequence, which was assumed to encode the function. However, the discovery of functional sequence variants proved that the functional encoding does not come down to the sequence, otherwise a change in the sequence would mean a change of function. Likewise, the discovery that function is fulfilled by a set of structures and not by a unique structure showed that the functional encoding does not come down to the structure either (Jaffe, 2005; Jaffe, 2020; Parisi et al., 2015).

The next alternative is that sets of atomic motions, achievable by different sequences and different structures, encode a specific function (Bahar et al., 2010). This is consistent with the multiple dynamic paths that fulfill allostery (Buchenberg et al., 2017). The challenge lies in distinguishing the set of dynamics associated with sequence variants functionally robust or functionally adapted (change of function) from the set of dynamics associated with functional failure. Inferring function from the protein dynamics is also important because pathological variants impacting the protein dynamics but not the protein structure, limit traditional structure-based drug discovery methods (Demir et al., 2021).

Network science is appropriate to study system dynamics from protein structures because it offers multiple avenues to study the complex spatiotemporal relationships between interacting entities (Barrat et al., 2004; Barabási, 2013; Unicomb et al., 2017). Integrative approaches combining experimental data or Molecular Dynamics (MD) simulation with network-based models enable to link protein structures to protein dynamics and function Demir et al. (2011), Leitner and Yamato (2018), Liang et al. (2018), Ponzoni and Bahar (2018), Bourgeat et al. (2019), Gheeraert et al. (2019), Melo et al. (2020), Bourgeat et al. (2021), Di Paola and Leitner (2021) and for a review see (Liang et al., 2020). On one hand, global mode analysis, elastic network models (ENM), dynamics network models (DNM) and protein energy networks (PEN) are used to track multiple scale dynamics in proteins, identify allosteric pathways and residues involved in biological activities. On the other, perturbation response scanning (PRS) and evolutionary network models are used to investigate the impact of mutations on protein features in particular for disease mutations. ELM applied on different protein family members also allows associating scale of motions to various types of activities (Wingert et al., 2021).

The advantages of network based models in probing protein dynamics come from the inference of amino acid and atomic links from the structure. Now, one on-going question is to clarify why network measures pinpoint functional residues (e.g., allostery) or distinguish disease mutations from the rest of the residues in order to better understand what properties amino acids have in a structure that make them functionally tolerant to mutations or not. The comparison of network measures and network models over proteins spanning large dynamics scales from enzyme to pore-forming toxins and over their sequence variants will help validating network measures as hallmarks of functional dynamics and of functional dynamic perturbations related to diseases.

One alternative perspective to network integrative approaches is to find network measures that are relevant to functional dynamics simply from protein structures. This implies a network measure probing collective slow motions and therefore shared across proteins and independent of amino acid features as observed from global modes (Bahar et al., 2010). In addition, a network measure with amino acid specific characteristics is expected if it embed the dynamics of a specific function. An allosteric enzyme and a pore-forming toxin have 3D-structures that share multiple scale collective dynamics but yet they have very different motions to fulfill their functions.

We consider the neighborhoods of each amino acid of a protein as potentially relevant to the problem. This is because on average over its neighbors every amino acid makes moderate and similar number of atomic interactions, a property shared by many different proteins (Dorantes-Gilardi et al., 2018). In addition, each neighborhood is different in terms of number of neighbors and type of neighbors (Dorantes-Gilardi et al., 2018). Thus, neighborhoods satisfy the two conditions to embed protein dynamics. Moreover, the neighborhoods describe the spatial position of the amino acids in the structure, which carves the space occupied by the amino acid atoms and hence uncovers the space left available between amino acids where atomic motions can take place. The relation between the space occupied by entities and the system dynamics is a broad topic from granular material to urban and protein systems (Liang and Dill, 2001; Majmudar and Behringer, 2005; Henzler-Wildman and Kern, 2007; Majmudar et al., 2007; Barthelemy, 2011; Dorantes-Gilardi et al., 2018; Gheeraert et al., 2019; Naganathan, 2019).

To analyze neighborhoods, a protein structure is modeled by an amino acid network (AAN), where the nodes are amino acids and the links are atomic interactions between amino acids, inferred from atomic proximity (Dorantes-Gilardi et al., 2018). The space occupied by neighborhoods is described in terms of amino acids with the node degree and in terms of atoms with the node weight (Methods). Classically, a unique cutoff around the threshold for chemical interactions (5 Å) is used to investigate protein features but here the neighborhoods are computed at different cutoff distances to probe the space occupancy at different spatial scales (Vuillon and Lesieur, 2015; Viloria et al., 2017). This condition is necessary to track the multiple dynamics scales associated with functional dynamics (Henzler-Wildman and Kern, 2007; Munoz and Cerminara, 2016).

Our case study is the third PDZ domain of the synaptic protein PDS-95 (PDB 1BE9) chosen because the functional impact of most of its single amino-acid mutations is known from experiments as well as some double mutations (McLaughlin et al., 2012; Salinas and Ranganathan, 2018). Thus, this case study is appropriate for future validation of the network measures to link dynamics and function.

## Methods

Starting from the Protein Data Bank (PDB) data, protein structures are modeled using the Amino Acid Network (AAN), an established model in Computational Biology (Dorantes-Gilardi et al., 2018). The AAN is a graph G = (V; E), with V is the set of the N nodes of the network (vertices of the graph) and E the set of links of the network (edges of the graph).

Nodes of the AAN: Each node in the AAN corresponds to one amino acid of the protein’s structure named according to the protein sequence:

V={i|i is an amino acid}. (1)

Links of the AAN: A link is an atomic interaction defined by atomic proximity: two amino acids *i* and *j* are connected if there exists at least one couple of atoms, one belonging to *i* and one belonging to *j*, at a distance lower or equal to a given threshold (Cutoff distance *c*),

$$\text{E}=\left\{\left(i,j\right)|\text{i},\text{j}\in \text{V}\;\text{with}\;\text{i}\neq \text{j}\;\text{and}\;\exists \left({\text{atom}}_{i}\in i,{\text{atom}}_{j}\in j\right)\text{with}\;\text{dist}\left({\text{atom}}_{i},{\text{atom}}_{j}\right)\leq c\right\}.$$ (2)

Link weights of the AAN: Each link is weighted according to the number of atomic couples that respect the cutoff condition:

$$w_{ij}=\left|\left\{\left({\text{atom}}_{i}\in i,{\text{atom}}_{j}\in j\right)\text{with}\;\text{dist}\left({\text{atom}}_{i},{\text{atom}}_{j}\right)\leq c\;\text{and}\;i\neq j\right\}\right|$$ (3)

where the pipe symbol | denotes the cardinality of the set (i.e. the number of elements of the set). When *c* = 5 Å, that is a threshold for chemical interactions, the link weights measure the number of atomic interactions between two amino acids.

Packing around amino acids: In the AAN, the node degree *k*_*i*_, defined as the number of amino acid neighbors of a node *i*, measures the amino-acid packing around the amino acid *i,* referred to as the amino-acid neighborhood. The node weight *w*_*i*_ is defined as the sum over all the weights of the links that connect the node *i* to its neighbors ( $w_{i}=\sum_{j\in N\left(i\right)}wij$ with *N(i)* the set of neighbors of node *i*). The node weight measures the atomic packing around the amino acid *i,* referred to as the atomic neighborhood.

Cutoff distance: Different cutoff distances are used in this study such that the packing around each amino acid is described at different length-scale via the neighborhoods at variable cutoffs. The cutoffs are integers and range from 3 to 11 Å such that the packing within chemical reach (≤5 Å) and above chemical reach (>5 Å) are monitored. The rational is to distinguish amino acids by their ‘chemical’ neighborhoods and above chemical reach neighborhoods to probe space occupancy involved in multiple spatiotemporal scales.

Plateau versus linear degree dependencies: The degree dependency with the cutoff is plotted. Some amino acids show a linear dependency of the degree with the cutoff and are referred to as linear amino acids. Some amino acids exhibit a plateau over some cutoffs determined with the derivative (here just Δk because the cutoffs are consecutive integers) equals to zero or equals to one if and only if the derivative at cutoffs before the plateau is equal to four or more. We refer to these amino acids as plateau amino acids.

Amino acid side chain length classification: Amino acids are classified by side-chain lengths as follows. Side chain length <3 Å are small amino acids (G, A, P, S, V, I, T, C), side chain length between 3 Å ≤ length <5 Å are medium amino acids (L, E, D, H, N, Q, M) and side chain length ≥5 Å are big amino acids (F, K, R, Y, W).

1D-barcode: The 1D-barcode represents the degree dependency with the cutoff (plateau- or linear-) of the amino acids of the 1BE9 sequence (1D).

2D-barcode: The 2D-barcode represents the degree dependency with the cutoff of the amino acids of the 1BE9 sequence (1D) at variable cutoffs (2D).

## Results and Discussion

The AAN of the PDZ domain of the synaptic protein PDS-95 is generated using the PDB 1BE9 and the weights and degrees of every amino acid nodes are computed at different cutoff distances (Methods). We recall that the weight of a node describes its atomic packing, referred to as its atomic neighborhood while the degree of a node describes its amino-acid packing referred to as its amino-acid neighborhood. The atomic neighborhood takes into account the features of the amino acids. The weight and degree cutoff dependencies are plotted to investigate how atoms and amino acid neighbors occupy the space around each amino acid at different scales in the protein structure. This is a proxy of the dynamics as the more space occupied the less space left available for atomic motions.

**FIGURE 1:**
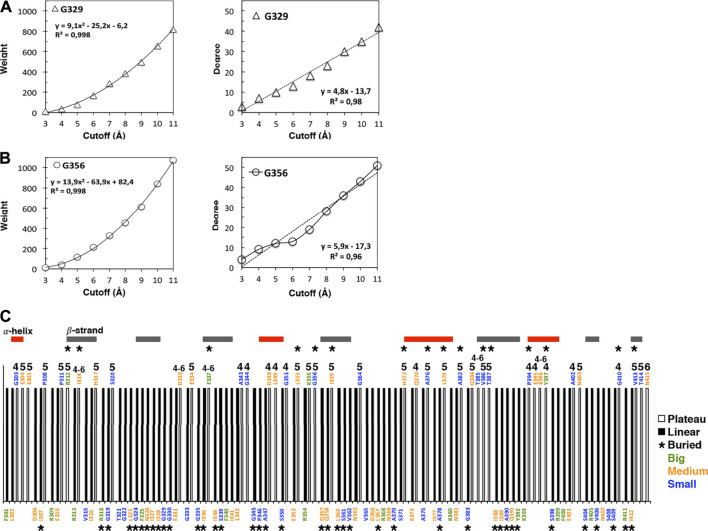
Cutoff dependencies of the weight and the degree of the AAN nodes of the 1BE9 structure. **(A)** Examples of the weight cutoff dependencies for glycine, the smallest amino acid. The increase is quadratic (R2 ∼1). **(B**) Example of the degree cutoff dependencies for the same amino acids. One is linear (R2 ∼0,98, **top panel**), and the other has a plateau between cutoffs 5 and 6 Å **(bottom panel)**. **(C)** 1D-barcode: cutoff dependencies of the 1BE9 sequence. The amino acids are colored according to the length of their extended side chains (blue: length <3 Å, orange: 3 Å ≤ length <5 Å, and green: length ≥5 Å). The colored horizontal bars represent the secondary structures along the sequence. The star is for buried amino acids, and the numbers indicate the cutoff range of the plateau: four is for a plateau at cutoffs 4 to 5 Å, 5 is for plateau at cutoffs five to 6 Å, six is for a plateau at cutoffs 6 to 7 Å and four–six is for a plateau at cutoffs 4 to 6 Å.

**FIGURE 2:**
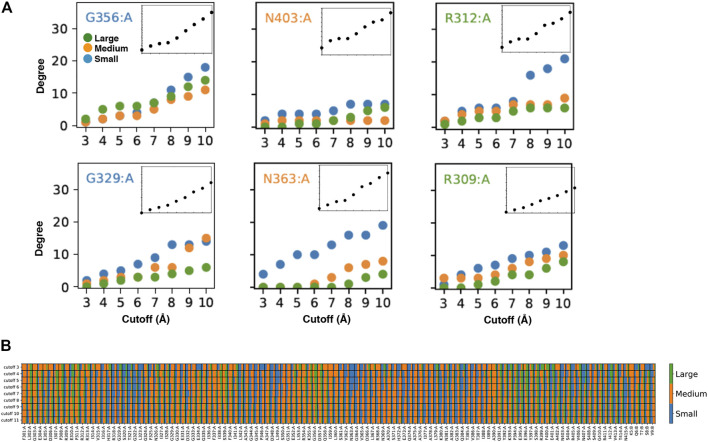
Customized amino acid neighborhoods. **(A)** Degree cutoff dependencies of the small, medium and big neighbors of the AAN nodes of the 1BE9 structure. The amino acids are colored according to the length of their extended side chains (blue: length <3 Å, orange: 3 Å ≤ length < 5 Å, green: length ≥ 5 Å). The Inset is the total degree cutoff dependency. Top row: neighbors sizes leading to plateau degree dependencies. Bottom row: neighbors sizes leading to linear degree dependencies **(B)** 2D-barcode. The percentage of small, medium and big side chain neighbors are indicated for each amino acid of the 1BE9 sequence (1D, horizontal axis) and at each cutoff (2D, vertical axis). On the sequence (horizontal), **(A**,**B)** stand for amino acids from the protein and the ligand, respectively.

The weight cutoff dependencies are quadratic indicating that the weight (the number of atomic interactions) increases with the square of the cutoff distance, i.e., with a surface of contact between atoms rather than a volume of contacts (Figure 1A; Supplementary Figure S1A). The degree cutoff dependencies are not quadratic but exhibit two distinct behaviors: neighborhoods with linear dependency with the cutoff (Figure 1B, top in the protein structure) and neighborhoods with a plateau over some cutoffs (Figure 1B, bottom and Supplementary Figure S1B). We call linear (plateau) amino acids, the amino acids whose degree follows a linear (plateau) dependency with the cutoff. The plateau is due to a lack of amino acid neighbors at higher cutoffs and not to a lack of atoms since the weight cutoff dependencies have no plateau (Supplementary Figure S1A). The plateau can result from having big amino acid neighbors that occupy the space over more than one cutoff versus having small amino acids that lead to a linear increase over the same cutoffs (Figure 2A).

Ten nodes have a plateau between 4 and 5 Å, twenty between 5 and 6 Å, one between 6 and 7 Å and six over 4 to 6 Å (Figure 1B).

All amino-acid types observed in 1BE9 except histidine which appears only twice, adopt plateau and linear neighborhoods (Figure 1C). Methionine, cysteine and tryptophan are not present in 1BE9. Twenty-six surface-exposed amino acids out of fifty-eight are plateau (∼half) and sixteen buried amino acids out of fifty-seven are plateau (∼a third). 27% of the β-strand amino acids are plateau against 35 and 48% for loops and α-helix amino acids, respectively. Thus the plateau and linear neighborhoods are achieved regardless amino-acid type, position in the structure and secondary structure, which makes the degree cutoff dependencies insensitive to amino acid features as global modes. Together with the plateau built at chemical-interaction threshold, it is consistent with the degree cutoff dependencies probing collective motions. Accordingly, a 1D-barcode representing the plateau- or linear-neighborhood of each amino acid of the sequence could be used to characterize the multiple scale dynamic features of a protein (Figure 1C).

To have a plateau or a linear degree cutoff dependency for one amino acid type implies customizing neighborhoods as illustrated by the higher number of big amino acid neighbors for plateau amino acids than linear amino acids (Figure 2A, compare top and bottom). To have it for any amino acid type also implies neighborhood customized to the central amino acids as seen on Figure 2A (compare across amino acid types). Thus, the linear and plateau neighborhoods accessible to all amino acids are nevertheless built from specific amino-acid neighborhoods (Figure 2A; Supplementary Figure S2). This means the protein dynamic-functional specificity could be embedded in the collective motions through specific space occupancy arising from neighborhood diversities. A 2D-barcode representing the amino acid neighborhood size specificities of each amino acid of the protein sequence (Figure 2B, horizontal axis) at different cutoffs (Figure 2B, vertical axis) could be used to characterize the protein specific embedded dynamics (Figure 2B).

We can see from this single case 2D barcode classes of neighborhoods in terms of neighbor sizes which anticipate classes of dynamics and of responses upon mutations supporting the possibility to use the data for mining dynamics and its relation to function (Figure 2B). Some positions are composed of a majority of one-size neighbors over the cutoffs (e.g. small neighbors: R318, T321, G322, L349, N363; medium: F301, G303, R312, G333, G351, A390) while others are a mixture of neighbor sizes (e.g. A308, I341, K355, A383, Q391, E401). In addition, some positions change neighbor sizes over the cutoffs (e.g., E396, E401). However, these sole data do not have the statistics to make hypothesis on which neighborhood classes lead to which dynamic classes or draw conclusion between the neighborhood specificity, dynamics and function.

## Conclusion

The study shows that amino acid neighborhoods and not only amino acids and amino acid pairs, contain information relevant to protein dynamics, opening a new perspective to explore the link between dynamics and function. The 1D barcode of, for example, an enzyme and a pore-forming toxin can be compared to determine common and distinct features which can in turn be analyzed with the 2D barcode to survey both the neighborhood diversity of the common 1D barcode features and of the distinct 1D barcode features assuming the former identifies positions functionally insensitive (protein aspecific) and the later positions functionally sensitive (protein specific). The analysis of each protein variants can be used to validate the assumption as well as database analysis.

This will contribute to diagnosing dynamic functional faults and dynamic functional diversity.

## Data Availability

The datasets presented in this study can be found in online repositories. The names of the repository/repositories and accession number(s) can be found in the article/Supplementary Material.

## References

[B1] Bahar I. Lezon T. R. Bakan A. Shrivastava I. H. (2010). Normal Mode Analysis of Biomolecular Structures: Functional Mechanisms of Membrane Proteins. Chem. Rev. 110 (3), 1463–1497. 10.1021/cr900095e 19785456 PMC2836427

[B2] Barabási A.-L. (2013). Network Science. Phil. Trans. R. Soc. A. 371 (1987), 20120375. 10.1098/rsta.2012.0375 23419844

[B3] Barrat A. Barthelemy M. Pastor-Satorras R. Vespignani A. (2004). The Architecture of Complex Weighted Networks. Proc. Natl. Acad. Sci. 101 (11), 3747–3752. 10.1073/pnas.0400087101 15007165 PMC374315

[B4] Barthelemy M. (2011). Spatial Networks. Phys. Rep. 499 (1–3), 1–101. 10.1016/j.physrep.2010.11.002

[B5] Bourgeat L. Pacini L. Serghei A. Lesieur C. (2021). Experimental Diagnostic of Sequence-Variant Dynamic Perturbations Revealed by Broadband Dielectric Spectroscopy. Structure. 10.1016/j.str.2021.05.005 34051139

[B6] Bourgeat L. Serghei A. Lesieur C. (2019). Experimental Protein Molecular Dynamics: Broadband Dielectric Spectroscopy Coupled with Nanoconfinement. Sci. Rep. 9 (1), 17988. 10.1038/s41598-019-54562-8 31784681 PMC6884508

[B7] Buchenberg S. Sittel F. Stock G. (2017). Time-resolved Observation of Protein Allosteric Communication. Proc. Natl. Acad. Sci. USA 114 (33), E6804–E6811. 10.1073/pnas.1707694114 28760989 PMC5565459

[B8] Demir Ö. Baronio R. Salehi F. Wassman C. D. Hall L. Hatfield G. W. (2011). Ensemble-based Computational Approach Discriminates Functional Activity of P53 Cancer and rescue Mutants. Plos Comput. Biol. 7 (10), e1002238. 10.1371/journal.pcbi.1002238 22028641 PMC3197647

[B9] Demir Ö. Barros E. P. Offutt T. L. Rosenfeld M. Amaro R. E. (2021). An Integrated View of P53 Dynamics, Function, and Reactivation. Curr. Opin. Struct. Biol. 67, 187–194. 10.1016/j.sbi.2020.11.005 33401096 PMC8985518

[B10] Di Paola L. Leitner D. M. (2021). Network models of biological adaptation at the molecular scale: Comment on" Dynamic and thermodynamic models of adaptation" by AN Gorban et al. Phys. Life Rev. 38, 124–126. 10.1016/j.plrev.2021.05.008 34090823

[B11] Dorantes-Gilardi R. Bourgeat L. Pacini L. Vuillon L. Lesieur C. (2018). In Proteins, the Structural Responses of a Position to Mutation Rely on the Goldilocks Principle: Not Too many Links, Not Too Few. Phys. Chem. Chem. Phys. 20 (39), 25399–25410. 10.1039/c8cp04530e 30272062

[B12] Gheeraert A. Pacini L. Batista V. S. Vuillon L. Lesieur C. Rivalta I. (2019). Exploring Allosteric Pathways of a V-type Enzyme with Dynamical Perturbation Networks. The J. Phys. Chem. B 123, 3452. 10.1021/acs.jpcb.9b01294 30943726 PMC6604606

[B13] Henzler-Wildman K. Kern D. (2007). Dynamic Personalities of Proteins. Nature 450 (7172), 964–972. 10.1038/nature06522 18075575

[B14] Jaffe E. K. (2020). Wrangling Shape-Shifting Morpheeins to Tackle Disease and Approach Drug Discovery. Front. Mol. Biosciences 7, 582966. 10.3389/fmolb.2020.582966 PMC7729013 33330623

[B15] Jaffe E. K. (2005). Morpheeins - a New Structural Paradigm for Allosteric Regulation. Trends Biochemical Sciences 30 (9), 490–497. 10.1016/j.tibs.2005.07.003 16023348

[B16] Leitner D. M. Yamato T. (2018). Mapping Energy Transport Networks in Proteins. Hoboken, New Jersey, US: Wiley Online Library.

[B17] Liang J. Dill K. A. (2001). Are Proteins Well-Packed? Biophysical J. 81 (2), 751–766. 10.1016/s0006-3495(01)75739-6 PMC1301551 11463623

[B18] Liang Z. Hu J. Yan W. Jiang H. Hu G. Luo C. (2018). Deciphering the Role of Dimer Interface in Intrinsic Dynamics and Allosteric Pathways Underlying the Functional Transformation of DNMT3A. Biochim. Biophys. Acta (Bba) - Gen. Subjects 1862 (7), 1667–1679. 10.1016/j.bbagen.2018.04.015 29674125

[B19] Liang Z. Verkhivker G. M. Hu G. (2020). Integration of Network Models and Evolutionary Analysis into High-Throughput Modeling of Protein Dynamics and Allosteric Regulation: Theory, Tools and Applications. Brief. Bioinformatics 21 (3), 815–835. 10.1093/bib/bbz029 30911759

[B20] Majmudar T. S. Sperl M. Luding S. Behringer R. P. (2007). Jamming Transition in Granular Systems. Phys. Rev. Lett. 98 (5), 058001. 10.1103/PhysRevLett.98.058001 17358902

[B21] Majmudar T. S. Behringer R. P. (2005). Contact Force Measurements and Stress-Induced Anisotropy in Granular Materials. Nature 435 (7045), 1079–1082. 10.1038/nature03805 15973358

[B22] McLaughlin R. N. Jr Poelwijk F. J. Raman A. Gosal W. S. Ranganathan R. (2012). The Spatial Architecture of Protein Function and Adaptation. Nature 491 (7422), 138–142. 10.1038/nature11500 23041932 PMC3991786

[B23] Melo M. C. R. Bernardi R. C. De La Fuente-nunez C. Luthey-Schulten Z. (2020). Generalized Correlation-Based Dynamical Network Analysis: a New High-Performance Approach for Identifying Allosteric Communications in Molecular Dynamics Trajectories. J. Chem. Phys. 153 (13), 134104. 10.1063/5.0018980 33032427

[B24] Muñoz V. Cerminara M. (2016). When Fast Is Better: Protein Folding Fundamentals and Mechanisms from Ultrafast Approaches. Biochem. J. 473 (17), 2545–2559. 10.1042/bcj20160107 27574021 PMC5003694

[B25] Naganathan A. N. (2019). Modulation of Allosteric Coupling by Mutations: from Protein Dynamics and Packing to Altered Native Ensembles and Function. Curr. Opin. Struct. Biol. 54, 1–9. 10.1016/j.sbi.2018.09.004 30268910 PMC6420056

[B26] Parisi G. Zea D. J. Monzon A. M. Marino-Buslje C. (2015). Conformational Diversity and the Emergence of Sequence Signatures during Evolution. Curr. Opin. Struct. Biol. 32, 58–65. 10.1016/j.sbi.2015.02.005 25749052

[B27] Ponzoni L. Bahar I. (2018). Structural Dynamics Is a Determinant of the Functional Significance of Missense Variants. Proc. Natl. Acad. Sci. U S A. 115, 4164–4169. 10.1073/pnas.1715896115 29610305 PMC5910821

[B28] Salamanca Viloria J. Allega M. F. Lambrughi M. Papaleo E. (2017). An Optimal Distance Cutoff for Contact-Based Protein Structure Networks Using Side-Chain Centers of Mass. Sci. Rep. 7 (1), 2838–2911. 10.1038/s41598-017-01498-6 28588190 PMC5460117

[B29] Salinas V. H. Ranganathan R. (2018). Coevolution-based Inference of Amino Acid Interactions Underlying Protein Function. eLife 7, e34300. 10.7554/eLife.34300 30024376 PMC6117156

[B30] Unicomb S. Iñiguez G. Karsai M. (2017). Threshold Driven Contagion on Weighted Networks. arXiv preprint arXiv:1707.02185. 10.1038/s41598-018-21261-9 PMC5814462 29449569

[B31] Vuillon L. Lesieur C. (2015). From Local to Global Changes in Proteins: a Network View. Curr. Opin. Struct. Biol. 31, 1–8. 10.1016/j.sbi.2015.02.015 25791607

[B32] Wingert B. Krieger J. Li H. Bahar I. (2021). Adaptability and Specificity: How Do Proteins Balance Opposing Needs to Achieve Function? Curr. Opin. Struct. Biol. 67, 25–32. 10.1016/j.sbi.2020.08.009 33053463 PMC8036234

