# Supplementary material for: Mapping Function from Dynamics: Future Challenges for Network-Based Models of Protein Structures
Source: Front Mol Biosci. 2021 Oct 11;8:744646. doi: 10.3389/fmolb.2021.744646 (PMC8543124; doi:10.3389/fmolb.2021.744646)
Supplement: Supplementary file 1 [file DataSheet1.pdf]

Supplementary Figure 1. Cutoff dependencies of the weight and the degree of the AAN nodes of the 1BE9 structure. **A.** Weight cutoff dependencies. **B.** Degree cutoff dependencies for the same amino acids. The amino acids are colored according to the length of their extended side chains (blue: length < 3 Å, orange: 3 Å ≤ length < 5 Å, green: length ≥ 5 Å).

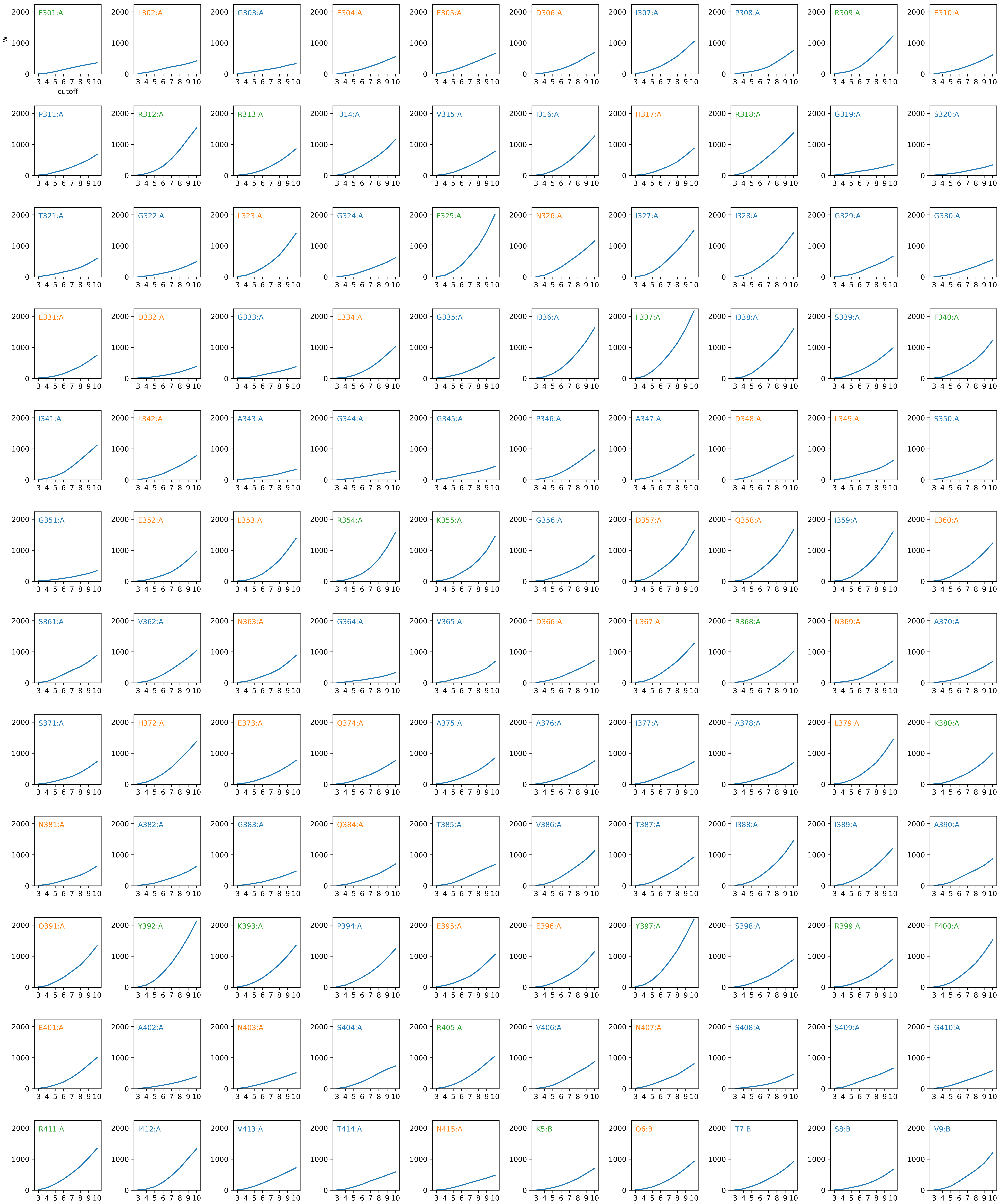
w
cutoff
F301:A
L302:A
G303:A
E304:A
E305:A
D306:A
I307:A
P308:A
R309:A
E310:A
P311:A
R312:A
R313:A
I314:A
V315:A
I316:A
H317:A
R318:A
G319:A
S320:A
T321:A
G322:A
L323:A
G324:A
F325:A
N326:A
I327:A
I328:A
G329:A
G330:A
E331:A
D332:A
G333:A
E334:A
G335:A
I336:A
F337:A
I338:A
S339:A
F340:A
I341:A
L342:A
A343:A
G344:A
G345:A
P346:A
A347:A
D348:A
L349:A
S350:A
G351:A
E352:A
L353:A
R354:A
K355:A
G356:A
D357:A
Q358:A
I359:A
L360:A
S361:A
V362:A
N363:A
G364:A
V365:A
D366:A
L367:A
R368:A
N369:A
A370:A
S371:A
H372:A
E373:A
Q374:A
A375:A
A376:A
I377:A
A378:A
L379:A
K380:A
N381:A
A382:A
G383:A
Q384:A
T385:A
V386:A
T387:A
I388:A
I389:A
A390:A
Q391:A
Y392:A
K393:A
P394:A
E395:A
E396:A
Y397:A
S398:A
R399:A
F400:A
E401:A
A402:A
N403:A
S404:A
R405:A
V406:A
N407:A
S408:A
S409:A
G410:A
R411:A
I412:A
V413:A
T414:A
N415:A
K5:B
Q6:B
T7:B
S8:B
V9:B

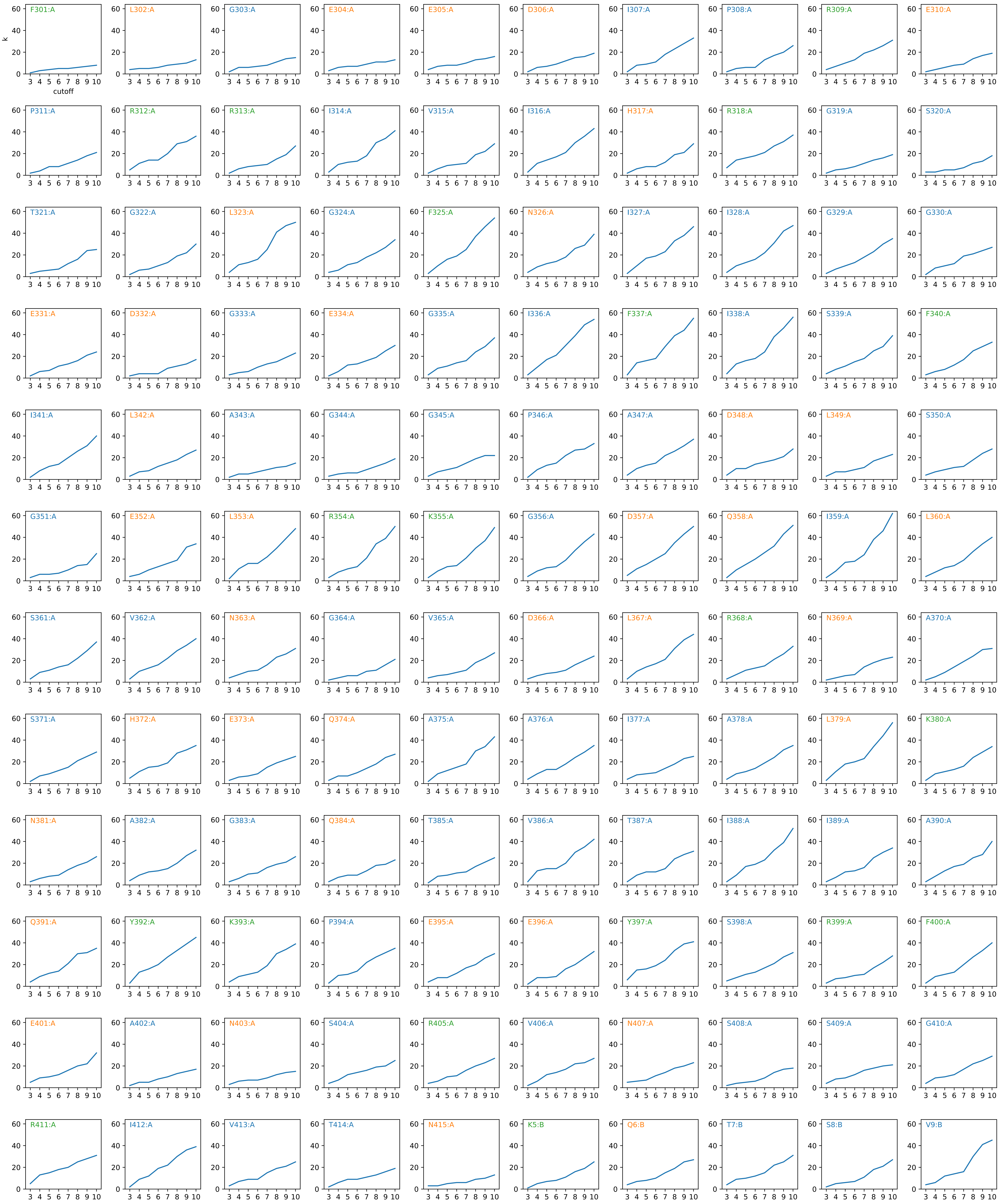

k
cutoff
0
20
40
60
3 4 5 6 7 8 9 10
F301:A
L302:A
G303:A
E304:A
E305:A
D306:A
I307:A
P308:A
R309:A
E310:A
P311:A
R312:A
R313:A
I314:A
V315:A
I316:A
H317:A
R318:A
G319:A
S320:A
T321:A
G322:A
L323:A
G324:A
F325:A
N326:A
I327:A
I328:A
G329:A
G330:A
E331:A
D332:A
G333:A
E334:A
G335:A
I336:A
F337:A
I338:A
S339:A
F340:A
I341:A
L342:A
A343:A
G344:A
G345:A
P346:A
A347:A
D348:A
L349:A
S350:A
G351:A
E352:A
L353:A
R354:A
K355:A
G356:A
D357:A
Q358:A
I359:A
L360:A
S361:A
V362:A
N363:A
G364:A
V365:A
D366:A
L367:A
R368:A
N369:A
A370:A
S371:A
H372:A
E373:A
Q374:A
A375:A
A376:A
I377:A
A378:A
L379:A
K380:A
N381:A
A382:A
G383:A
Q384:A
T385:A
V386:A
T387:A
I388:A
I389:A
A390:A
Q391:A
Y392:A
K393:A
P394:A
E395:A
E396:A
Y397:A
S398:A
R399:A
F400:A
E401:A
A402:A
N403:A
S404:A
R405:A
V406:A
N407:A
S408:A
S409:A
G410:A
R411:A
I412:A
V413:A
T414:A
N415:A
K5:B
Q6:B
T7:B
S8:B
V9:B

Supplementary Figure 2. Customized amino acid neighborhoods. Degree cutoff dependencies of the small (s), medium (m) and large (l) neighbors of the AAN nodes of the 1BE9 structure. The amino acids are colored according to the length of their extended side chains (blue: length < 3 Å, orange: 3 Å ≤ length < 5 Å, green: length ≥ 5 Å).

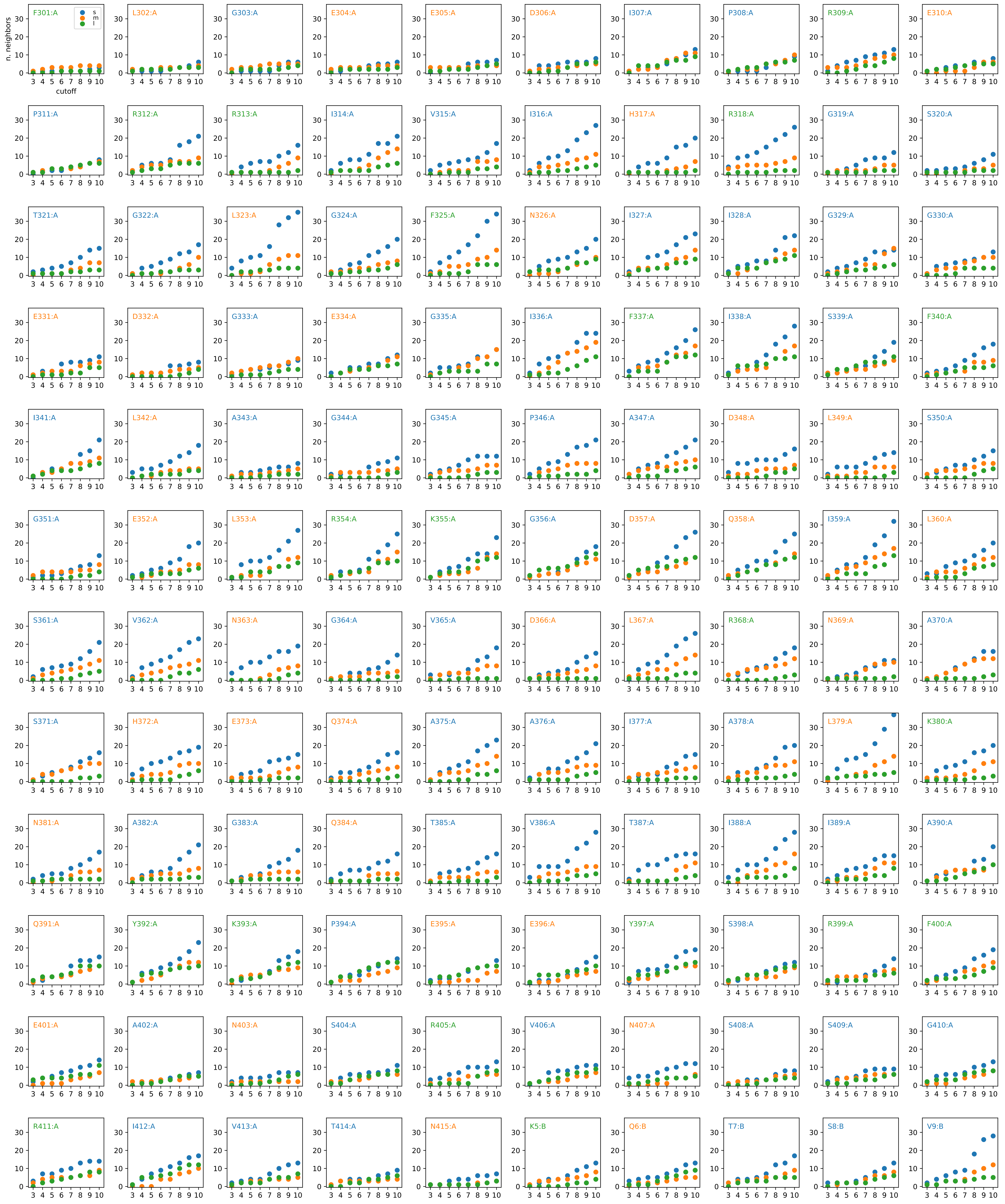
n. neighbors
cutoff
s
m
l
F301:A
L302:A
G303:A
E304:A
E305:A
D306:A
I307:A
P308:A
R309:A
E310:A
P311:A
R312:A
R313:A
I314:A
V315:A
I316:A
H317:A
R318:A
G319:A
S320:A
T321:A
G322:A
L323:A
G324:A
F325:A
N326:A
I327:A
I328:A
G329:A
G330:A
E331:A
D332:A
G333:A
E334:A
G335:A
I336:A
F337:A
I338:A
S339:A
F340:A
I341:A
L342:A
A343:A
G344:A
G345:A
P346:A
A347:A
D348:A
L349:A
S350:A
G351:A
E352:A
L353:A
R354:A
K355:A
G356:A
D357:A
Q358:A
I359:A
L360:A
S361:A
V362:A
N363:A
G364:A
V365:A
D366:A
L367:A
R368:A
N369:A
A370:A
S371:A
H372:A
E373:A
Q374:A
A375:A
A376:A
I377:A
A378:A
L379:A
K380:A
N381:A
A382:A
G383:A
Q384:A
T385:A
V386:A
T387:A
I388:A
I389:A
A390:A
Q391:A
Y392:A
K393:A
P394:A
E395:A
E396:A
Y397:A
S398:A
R399:A
F400:A
E401:A
A402:A
N403:A
S404:A
R405:A
V406:A
N407:A
S408:A
S409:A
G410:A
R411:A
I412:A
V413:A
T414:A
N415:A
K5:B
Q6:B
T7:B
S8:B
V9:B
